# Supplementary material for: Resurgence of Chloramphenicol Resistance in Methicillin-Resistant Staphylococcus aureus Due to the Acquisition of a Variant Florfenicol Exporter (fexAv)-Mediated Chloramphenicol Resistance in Kuwait Hospitals
Source: Antibiotics (Basel). 2021 Oct 15;10(10):1250. doi: 10.3390/antibiotics10101250 (PMC8532628; doi:10.3390/antibiotics10101250)
Supplement: Supplementary file 1 [file antibiotics-10-01250-s001.zip › antibiotics-1393410-supplementary.pdf]

antibiotics

MDPI

Supplementary

Resurgence of Chloramphenicol Resistance in Methicillin-Resistant *Staphylococcus aureus* Due to the Acquisition of a Variant Florfenicol Exporter (*fexAv*)-Mediated Chloramphenicol Resistance in Kuwait Hospitals

Edet E. Udo *, Samar S. Boswihi, Bindu Mathew, Bobby Noronha, and Tina Verghese

Department of Microbiology, Faculty of Medicine, Kuwait University; Kuwait City, Kuwait; samar.boswihi@ku.edu.kw (S.S.B.); bindujmk@gmail.com (B.M.); bobby.vaz@gmail.com (B.N.); tinajay@gmaail.com (T.V.)

* Correspondence: udo.ekpenyong@ku.edu.kw; Tel.: +965-99553167

Table S1. Virulence gene profile of chloramphenicol-resistant MRSA isolates.

Virulence Factors	ST627-VI-t688 (MRSA-VI +SCCfus)	ST5-V-t688 (WA MRSA 11/34)	ST5-V-t688 (WA MRSA 81/85)	ST627-VI-t450 (MRSA-VI +SCCfus)	ST627-VI-t954 (MRSA-VI +SCCfus)	ST239-III-t860 (Vienna/Brazilian)	ST239-III-t037 (Vienna/Brazilian)
**Regulatory Genes**							
sarA	+	+	+	+	+	+	+
saes	+	+	+	+	+	+	+
agrI	-	-	-	-	-	+	+
agrII	+	+	+	+	+	-	-
**Enterotoxins**							
sea	+	+	+	+	+	-	+
sed	+	+	-	+	+	-	-
sej	+	+	-	+	+	-	-
sek	-	-	-	-	-	+	+
seq	-	-	-	-	-	+	+
ser	+	+	-	+	+	-	-
egc	+	+	+	+	+	-	-
**Leukocidins**							
lukF	+	+	+	+	+	+	+
lukS	+	+	+	+	+	+	+
lukD	+	+	+	+	+	+	+
lukE	+	+	+	+	+	+	+
lukX	+	+	+	+	+	+	+
lukY	+	+	+	+	+	+	+
**Hemolysins**							
hlgA	+	+	+	+	+	+	+
hld	+	+	+	+	+	+	+
hl	+	+	+	+	+	+	+
hla	+	+	+	+	+	+	-
hlb	+	+	+	+	+	+	+
hllll	+	+	+	+	+	+	+
			**Hlb-Converting Phages**				
Sak	+	+	+	+	+	+	+
Chp	-	-	-	-	-	+	-
Scn	+	+	+	+	+	+	+

			**Capsular Polysaccharide Associated Genes**				
Cap 5	+	+	+	+	+	-	-
Cap 8	-	-	-	-	-	+	+
**Virulence Factors**	ST627-VI-t688 (MRSA-VI +SCCfus)	ST5-V-t688 (WA MRSA 11/34)	ST5-V-t688 (WA MRSA 81/85)	ST627-VI-t450 (MRSA-VI +SCCfus)	ST627-VI-t954 (MRSA-VI +SCCfus)	ST239-III-t860 (Vienna/Brazilian)	ST239-III-t037 (Vienna/Brazilian)
**Proteases**							
Aur	+	+	+	+	+	+	+
splA	+	+	+	+	+	-	+
splB	+	+	+	+	+	-	+
splE	-	-	-	-	-	-	+
sspA	+	+	+	+	+	+	+
sspB	+	+	+	+	+	+	+
**Adhesion Factors**							
clfA	+	+	+	+	+	+	+
clfB	+	+	+	+	+	+	+
cna	-	-	-	-	-	+	+
Fib	+	+	+	+	+	+	+
fnbA	+	+	+	+	+	+	+
fnbB	+	+	+	+	+	+	+
map	+	+	+	+	+	+	+
sasG	+	+	+	+	+	+	+
			**Biofilm Associated Genes**				
icaA	+	+	+	+	+	+	+
icaC	+	+	+	+	+	+	+
icaD	+	+	+	+	+	+	+
			**Type1 Restriction Modification System**				
hsdS1							
hsdS2	-	+	+	-	-	+	+
hsdS3	+	+	+	+	+	-	+
hsdSx (CC25)	-	+	+	+	+	+	+
hsdSx (CC15)	+	-	-	-	-	-	-

Abbreviations: aur, aureolysin, hlgA, haemolysin gamma; hla, haemolysin alpha; hlb, haemolysin beta; hl/hlIII, putative membrane protein; sak, staphylokinase; chp, chemotaxis-inhibiting protein; scn, staphylococcal complement inhibitor, clfA, clumping factor A; clfB, clumping factor B; fnbA, fibronectin-binding protein A; fnbB, fibronectin-binding protein map, major histocompatibility complex class II; cna, collagen-binding adhesin. SplA, serinprotease A, SplB, serinprotease B, SplE, serinprotease E, sspA, glutamylendopeptidase, sspB, staphopain B, protease. Enterotoxin gene cluster (egc) consist of *seg, sei, selm, seln, selo, selu.* All isolates were negative for genes encoding Panton Valentine leucocidin (PVL), toxic shock syndrome toxin 1 (TSST1), exfoliative toxin (ET), Arginine Catabolic Mobile Element (ACME) and epidermal cell differentiation inhibitors (edin).
